# New Aspects of HERG K^+^ Channel Function Depending upon Cardiac Spatial Heterogeneity

**DOI:** 10.1371/journal.pone.0072181

**Published:** 2014-01-27

**Authors:** Pen Zhang, Ping Guan, Xiao-Lu Bai, Zhi-Ping Song

**Affiliations:** Department of Cardiology, Minhang Central Hospital, Shanghai, China; University of Tampere, Finland

## Abstract

HERG K^+^ channel, the genetic counterpart of rapid delayed rectifier K^+^ current in cardiac cells, is responsible for many cases of inherited and drug-induced long QT syndromes. HERG has unusual biophysical properties distinct from those of other K^+^ channels. While the conventional pulse protocols in patch-clamp studies have helped us elucidate these properties, their limitations in assessing HERG function have also been progressively noticed. We employed AP-clamp techniques using physiological action potential waveforms recorded from various regions of canine heart to study HERG function in HEK293 cells and identified several novel aspects of HERG function. We showed that under AP-clamp I_HERG_ increased gradually with membrane repolarization, peaked at potentials around 20–30 mV more negative than revealed by pulse protocols and at action potential duration (APD) to 60%-70% full repolarization, and fell rapidly at the terminal phase of repolarization. We found that the rising phase of I_HERG_ was conferred by removal of inactivation and the decaying phase resulted from a fall in driving force, which were all determined by the rate of membrane repolarization. We identified regional heterogeneity and transmural gradient of I_HERG_ when quantified with the area covered by I_HERG_ trace. In addition, we observed regional and transmural differences of I_HERG_ in response to dofetilide blockade. Finally, we characterized the influence of HERG function by selective inhibition of other ion currents. Based on our results, we conclude that the distinct biophysical properties of HERG reported by AP-clamp confer its unique function in cardiac repolarization thereby in antiarrhythmia and arrhythmogenesis.

## Introduction

Cardiac function, in particular the cardiac electrical activity, is controlled by an array of ion channels that activate in a well-organized manner with a delicate balance between inward and outward ion movements. Upon receiving an incoming impulse, cardiac cells excite with a rapid membrane depolarization followed by a relatively slow repolarization process. Repolarization abnormalities, either excessive slowing or accelerating of the rate of repolarization, can cause cardiac electrical perturbations or arrhythmias. The rate of repolarization is determined by a team effort of several ion currents, of which the rapid delayed rectifier K^+^ current (I_Kr_) is a crucial player, particularly to the plateau phase of an action potential [1]–[3]. The major molecular component of native I_Kr_ has been identified as the human *ether-a-go-go*-related gene (*HERG*) which when expressed in heterologous systems generates I_Kr_-like current [4]. Impairment of I_Kr_/HERG can cause excessive prolongation of action potential duration (APD). Indeed, HERG has been implicated as a molecular target for mutations leading to induction of chromasome-7 linked type 2 familial long QT syndrome and as a pharmacological target for drug actions that induce acquired long QT syndrome, a lethal ventricular tachyarrhythmia often leading to sudden cardiac death [4].

HERG possesses unique biophysical characteristics and pharmacological properties distinct from other K^+^ channels. During membrane depolarization, HERG K^+^ channel simultaneously activates and inactivates; but its inactivation kinetics is faster than its activation kinetics, with inactivation time constants (in the order of ms) some 1–2 orders of magnitude smaller than the activation time constants (in a range up to hundreds of ms) at the same potential. Upon membrane repolarization or hyperpolarization, HERG channel rapidly reactivates from inactivated state and slowly deactivates; the reactivation kinetics is similar to the inactivation time course whereas the deactivation process is approximately two orders of magnitude slower than the inactivation, at the same membrane potential. These biophysical characteristics confer HERG a strong inward rectification and a bell-shaped current-voltage relationship (I–V relationship). Presumably due to its wide-opened pore architecture, HERG channel is susceptible to blockade by a variety of drugs and compounds of various categories with heterogeneous structures [5]. This high pharmacological sensitivity renders HERG the most frequent cause of drug-induced long QT syndrome. In addition, HERG might also be involved in pathological QT prolongation. Heart failure is electrophysiologically characterized by abnormal QT prolongation and early afterdepolarization (EAD) [6]. Indeed, depression of I_Kr_ has been documented in failing hearts of both animal models [7], [8] and theoretical simulation [9]. Tumor necrosis factor-α (TNF-α), a critical factor causing heart failure and when overexpressed prolonging APD and promoting ventricular arrhythmias, suppresses HERG function [10]. Recent studies further suggest that decreasing I_Kr_ increases rate-related APD alternants that can dispose to ventricular tachyarrhythmias [11], [12]. On the other hand, enhancement of HERG function might also be proarrhythmic. A study demonstrated that I_Kr_-like current is increased in cells from acute myocardial infarction that is associated with arrhythmias [13], [14]. And it has been shown that HERG function is enhanced by phospholipid metabolites that are known to accumulate during the early phase of acute myocardial ischemia and to cause ischemic arrhythmias by shortening APD [10], [15]–[17]. These data indicate that I_Kr_/HERG plays an important role in determining propensity of arrhythmias. Understanding HERG function is therefore crucial to understanding mechanisms of arrhythmias of various types under different pathological conditions or induced by drug toxicity.

A cardiac action potential (AP) is typically divided into phase 0 depolarization, phase 1 repolarization, phase 2 plateau, and phase 3 repolarization, with magnitude, length and rate of these phases varying in cells from different regions of the heart. The key feature of cardiac AP is its relatively long repolarization process, ranging from decades of to hundreds of mille-seconds depending on species and regions from which the cells are investigated, compared to its rapid and brief phase 0 depolarization (within 2 ms). This implies that during each excitation a cardiac cell spends a majority of time in the repolarization process after its peak depolarization. Another important note about the cardiac APs is the variability of AP waveforms; the regional differences (such as sinus-atrial nodal (SAN), atrial and ventricular cells) and transmural heterogeneity (such as endocardium, midmyocardium and epicardium of ventricular walls) [18]–[20]. Noticeably, given the unique features of voltage- and time-dependence of HERG channel and the characteristics of cardiac APs, HERG channel activities are expected to differ during membrane repolarization from during depolarization, and HERG function assessed with repolarizing waveforms (or AP configurations) should be of more physiological relevance. However, HERG function has been studied primarily by conventional voltage step protocols with long rectangular depolarizing pulses of usually ≥2-s in duration–non-physiological voltage waveforms. This view raises a serious doubt about the fidelity of depolarizing pulses to reflect the true physiological function and pharmacological sensitivity of HERG K^+^ channels. Some efforts have been made in previous studies to address this apparent problem by using either authentic AP waveforms or simulated AP configurations in assessing HERG function [11], [12], [21]–[23]. These studies indeed revealed some important features of HERG channels, which are otherwise masked by conventional square pulse protocols. Nonetheless, precise understanding of HERG activity under more physiological conditions during an AP is still lacking. Specifically, several unanswered questions await more detailed investigation. First, what are the biophysical attributes that determine HERG function during an AP in cardiac cells? Or conversely, how do changes of HERG function influence cardiac repolarization? Second, whether there exist regional differences in terms of HERG function and if yes how do these differences contribute to the highly organized cardiac repolarization? Finally, how does I_Kr_/HERG change in response to inhibition of ion currents that activate before I_Kr_/HERG does? The present study was designed to delineate these points with the use of AP-clamp techniques.

## Methods

### Cell Culture

HEK293 cells stably expressing HERG were grown in Dulbecco's modified Eagle's medium (DMEM) supplemented with 10% heat-inactivated fetal bovine serum, 200 µM G418, 100 units/ml penicillin, and 100 µg/ml streptomycin. For electrophysiological study, cells subcultured to ∼85% confluency were harvested by trypsinization and stored in the Tyrode solution containing 0.5% bovine serum albumin at 4°C [24]. Cells were studied within 10 hours of harvest.

### Myocyte Isolation

For action potential clamp (AP-clamp) experiments, APs were obtained from myocytes isolated from different regions of canine hearts. The procedure for canine myocyte isolation was essentially the same as described in detail elsewhere [25]. Briefly, adult male mongrel dogs were anesthetized with morphine (2 mg/kg SC) and α-chloralose (120 mg/kg IV load, 29.25 mg/kg/h infusion), and a median sternotomy was performed. The hearts were removed and mounted to a Langendorff perfusion system. The preparation was perfused with Ca^2+^-containing Tyrode solution at 37°C until the effluent was clear of blood, and the perfusate was then switched to Ca^2+^-free Tyrode solution for 20 min at a constant rate of 25 ml/min, followed by perfusion with the same solution containing collagenase and 0.1% BSA for 35 min. The right atrium was dissected out in Kraftbrühe (KB) medium and cells were dispersed by agitation. Dissection of different layers of left ventricular wall was carried out according to the method established by Liu *et al* [26]. Briefly, after perfusion, thin slices of tissues were dissected from epicardium (<1.5 mm from epicardial surface), M region (3 to 5 mm from epicardial surface), and endocardium (<2 mm from the endocardial surface). Shavings were made parallel to the surface of the left ventricular free wall midway along the apicobasal axis. The SAN region was dissected out and cut into small pieces in the enzyme solution for continuous digestion for another 20 min. Cells were then stored in KB medium for later use. A total of six adult male mongrel dogs (22∼27 kg) were used in this study. The protocols for the use of dogs in this study were approved by the Animal Use and Care Committee of the Minhang Central Hospital.

### Whole-Cell Patch-Clamp Recording

Patch-clamp recording of HERG K^+^ currents (I_HERG_) was conducted with the whole-cell voltage-clamp methods in the voltage-clamp mode, and single cell action potential was recorded in the current-clamp mode, using an Axopatch 200B amplifier (Axon Instruments, Burlingame, CA). Borosilicate glass electrodes (1 mm) had tip resistances of 1 to 3 MΩ when filled with pipette solution containing (mM) 130 KCl, 1 MgCl_2_, 5 Mg-ATP, 10 EGTA, and 10 HEPES (pH 7.3). Junction potentials were zeroed before formation of the membrane-pipette seal in the extracellular (Tyrode) solution containing (mM) 136 NaCl, 5.4 KCl, 1 CaCl_2_, 1 MgCl_2_, 10 glucose, and 10 HEPES (pH 7.4). Single cell action potentials (APs) in myocytes isolated from various regions of canine hearts were revoked by 10 consecutive stimuli of 3-ms duration and twice threshold strength at a frequency of 1 Hz (or a cycle length of 1 s). The APs recorded by the 10^th^ pulse reached the steady state and were converted to pCLAMP 6 format for use as voltage-clamp waveforms using the DacFile command in pCLAMP 6. For current recordings in HERG-expressing HEK293 cells, the capacitance and series resistance were electrically compensated to minimize the duration of the capacitative surge on the current recording and the voltage drop across the clamped cell membrane. Cells with changing leak current (indicated by >10 pA changes in holding current at 250 mV) were rejected. Experiments were conducted at 36±1°C. All chemicals were purchased from Sigma Chemical Co.

### Data Analysis

Group data are expressed as mean ± S.E. Statistical comparisons (performed using ANOVA followed by Dunnett's method) were carried out, and paired or unpaired *t*-test was used, as appropriate, for single comparisons, using Microsoft Excel. A two-tailed *p*<0.05 was taken to indicate a statistically significant difference. Nonlinear least square curve fitting was performed with CLAMPFIT in pCLAMP 8.0 or GraphPad Prism and integration was performed to calculate the area under I_HERG_ trace using GraphPad Prism.

## Results

### Characteristics of Action Potentials from Various Regions of the Heart

**Figure 1:**
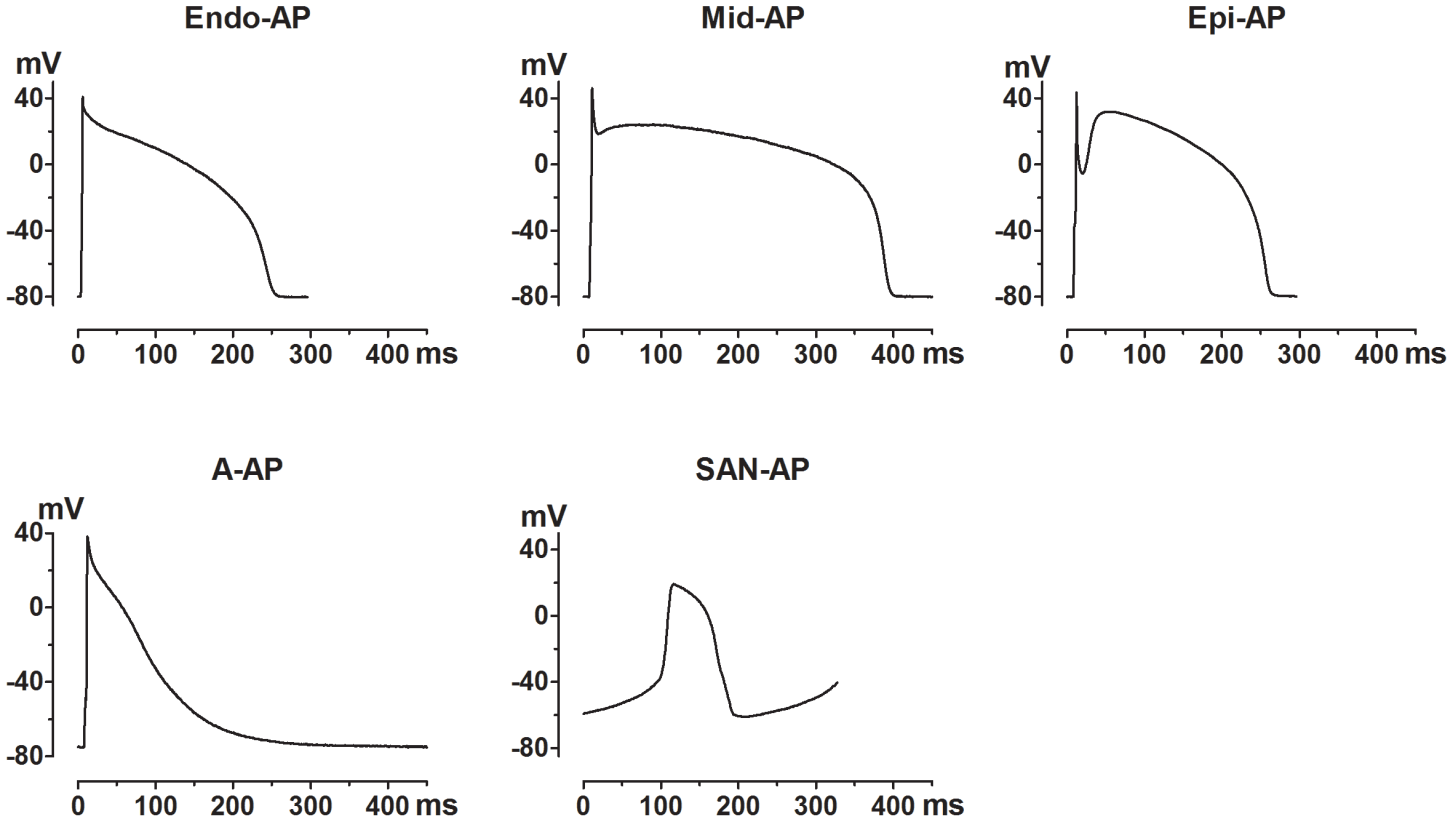
Action potential (AP) waveforms recorded from myocytes isolated from various regions of the canine heart. These AP waveforms were used for the AP-clamp studies of HERG K^+^ channel function in HERG-expressing HEK293 cells. The quantitative characteristics of these APs are summarized in Table 1.

Action potentials (APs) were recorded in cardiomyocytes isolated from various regions of canine hearts, including endocardium, midmyocardium and epicardium of left ventricular wall, right atrium and SAN. APs from different regions had different morphology (Fig. 1) and characteristics (Table 1).

**Table 1:** Characteristics of Action Potentials Recorded From Various Regions of Canine Heart and Used as Command Waveforms for AP-Clamp.

	Endo-AP	Mid-AP	Epi-AP	A-AP	SAN-AP
**APD_10_ (mV—ms)**	29—15	34—13	31—13	27—17	11—44
**APD_20_ (mV—ms)**	17—65	21—155	19—32	16—30	3—58
**APD_50_ (mV; ms)**	−20—197	−17—366	−18; 230	−19; 82	−21; 72
**APD_60_ (mV—ms)**	−32—219	−30—378	−31—245	−30—96	−29—75
**APD_70_ (mV—ms)**	−44—232	−42—384	−43—249	−46—135	−41—80
**APD_90_ (mV—ms)**	−68—246	−68—390	−68—257	−64—179	−53—89
**RR2 (mV/sec)**	39	22	36	45	40
**RR3 (mV/sec)**	72	80	75	35	65
**RR4 (mV/sec)**	72	80	75	35	65
**SDR (mV/sec)**	N/A	N/A	−65	N/A	N/A

**APD_10_**……. **APD_90_**, action potential duration at 10%.........90% full repolarization, respectively; **RR2**, **RR3** and **RR4**, repolarization rates corresponding to the phases of APD_50_, APD_50_-APD_60_ and APD_60_-APD_90_, respectively; **SDR**, secondary depolarization rate.

The AP from epicardium (Epi-AP) was characterized by prominent spike-and-dome morphology, with an initial rapid and pronounced repolarization followed by a secondary depolarization. Midmyocardial AP (Mid-AP) demonstrated a moderate phase 1 spike and a long flat phase 2 plateau. The AP of endocardial cells (Endo-AP) has a noticeable phase 1 repolarization and steeper phase 2 repolarization. The APs from three layers of left ventricular wall all had a rapid and regenerative phase 4 repolarization. By comparison, typical atrial AP (A-AP) was characterized by a short plateau phase and a slow final repolarization, approximating triangular configuration. The AP from sinus-atrial nodal cells (SAN-AP) typically displayed a slow spontaneous depolarization followed by a rapid depolarization and short action potential duration (APD). To quantitatively characterize the APs, several parameters important for analyzing HERG function were measured and are summarized in Table 1. APD_10_, APD_20_, APD_50_, APD_60_, and APD_90_ were determined for APD to 10%, 20%, 50%, 60% and 90% of repolarization, respectively, and the voltages at the respective durations. AP amplitude (APA) and diastolic membrane potential (DMP) were measured as total amplitude of APs and voltage at diastolic interval, respectively. The rates of membrane repolarization for phase 2, phase 3 and phase 4 (RR2, RR3, and RR4, respectively) were determined by linear regressions to the steepest portion of the corresponding repolarizing phases, and the rate of secondary depolarization (SDR) was also calculated.

The rate of initial repolarization (APD_10_) was similar between various layers of ventricle and atrium, but was considerably slower in SAN. The transmural difference became prominent from the subsequent phases of repolarization. For instance, APD_20_ of Mid-AP was around 2.5-fold and 5-fold longer than Endo-AP and Epi-AP, respectively, in the order of Mid-AP>Endo-AP>SAN-AP>Epi-AP = A-AP. The length of Epi-AP began to exceed that of Endo-AP from phase 2 repolarization; the order of the length of APD_50_, APD_60_, and APD_90_ was Mid-AP>Epi-AP>Endo-AP>A-AP>SAN-AP. RR_1_. APA and DMP were in the same range for V-APs and A-AP, and lower for SAN-AP.

### Characteristics of HERG K^+^ channel kinetics by conventional pulse protocols

**Figure 2:**
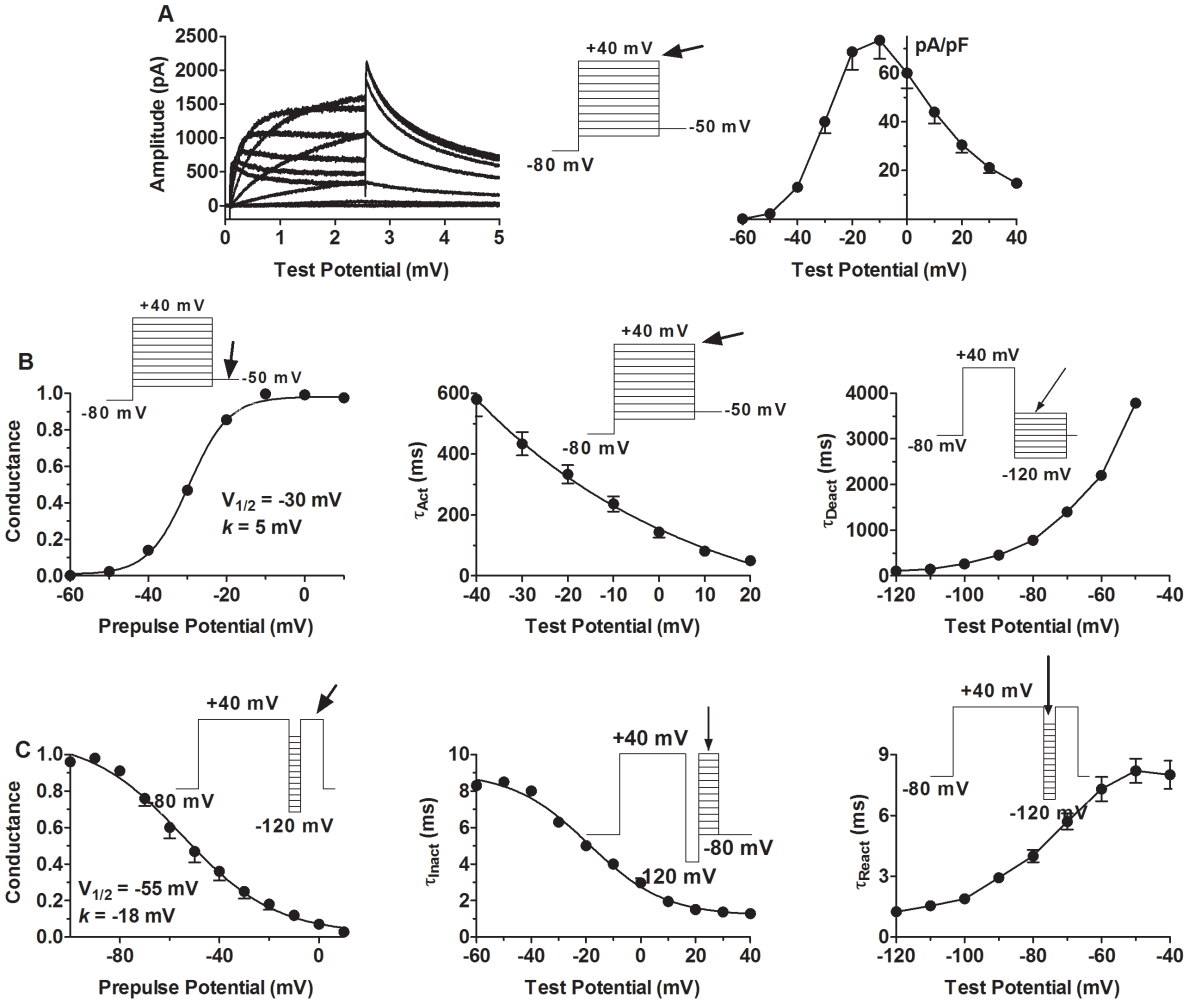
Characterization of I_HERG_ with conventional square-pulse protocols. ***A***, left panel: a typical example of I_HERG_ currents recorded with the voltage protocol shown in the inset; right panel: the averaged current density-voltage relationship of I_HERG_ (n = 12).. ***B***, left: steady-state voltage-dependent activation curves. The activation curves were constructed by plotting the conductance *G* as a function of depolarizing potentials. *G* was calculated by normalizing the tail currents at −50 mV by dividing the amplitude of the tail currents measured at various antecedent depolarizing potentials by that of the tail current at +40 mV. Symbols are mean of experimental data and lines represent the Boltzmann fit: *G/G_max_* = 1/{1+exp[(*V_1/2_−V*)/*k*]}, where *G_max_* represents the maximal conductance at +40 mV, *V_1/2_* is a half-maximal activation voltage, and *k* is a slope factor. Middle: the apparent activation time constants (τ_act_) of HERG channels were determined by the single exponential fit to the step I_HERG_ during depolarizing pulse to 0 mV. Right: the apparent deactivation time constants (τ_deact_) of HERG channels. The decaying phase of the tail I_HERG_ elicited at various hyperpolarizing/repolarizing steps (from −120 mV to −50 mV), following a depolarizing prepulse to +40 mV, were fit by the single exponential to obtain τ_deact_. ***C***, left: the steady-state voltage-dependent inactivation was assessed by the voltage protocol shown in the inset. The steady-state inactivation curves were constructed by plotting the channel availability or conductance *G* as a function of hyperpolarizing potentials. *G* was calculated by normalizing the tail currents elicited at +20 mV by dividing the amplitude of the tail currents measured at various antecedent hyperpolarizing potentials by that of the tail current at −100 mV. Symbols are the mean of experimental data and lines represent the Boltzmann fit: *G*/*G*_max_ = 1/{1+exp[(*V*_1/2_−*V*)/*k*]}, where *G*_max_ represents the maximal channel availability at −100 mV, *V*_1/2_ is the half-maximal inactivation voltage, and *k* is the slope factor. Middle: the inactivation kinetics (τ_inact_) of HERG channels. The decaying tail currents recorded upon depolarization to various potentials ranging from −80 mV to +40 mV to inactivate HERG channels, preceded by a 10-ms hyperpolarizing prepulse to −120 mV to let channels fully recover, were fit to the monoexponential function to obtain the values of τ_inact_. Right: the reactivation time course (τ_react_) determined with the same voltage protocol as that for steady-state inactivation. The monoexponential fit was applied to the activating phase of the tail I_HERG_ evoked at various hyperpolarizing/repolarizing steps following the inactivation steps at +40 mV. *p<0.05 *vs.* Ctl; n = 12 cells.

To aid understanding I_HERG_ function and biophysics with AP-clamp, comparison with the conventional pulse protocol was made. Depolarizing voltage steps from −60 mV to +40 mV elicited delayed rectifier outward currents with characteristic inward rectification that was manifested at stronger depolarization. Upon repolarization back to −50 mV, slowly decaying tail currents were recorded. The inward rectification resulted in a bell-shaped I–V relationship with I_HERG_ peaking at −15 mV (Fig. 2A).

#### Steady-state voltage-dependent activation

The steady-state voltage-dependent activation was determined by tail currents elicited by the voltage protocol shown in the inset of Fig. 2B. The half-maximal inactivation voltage (*V*_1/2_) and the slope factor (*k*) determined by the Boltzmann distribution were −30±2 mV and −5 mV, respectively.

#### Steady-state voltage-dependent inactivation

The steady-state voltage-dependent inactivation was assessed by the voltage protocol shown in the inset of Fig. 2C. I_HERG_ was first inactivated by a 2-s depolarizing step to +40 mV and then reactivated to various extents during the hyperpolarizing pulses to various potentials of a 10-ms duration that allowed for full reactivation with minimal deactivation, and the decaying outward currents induced by the subsequent 50 ms depolarizing pulse to +20 mV represent I_HERG_ inactivation. The half-maximal inactivation voltage (*V*_1/2_) and the slope factor (*k*) determined by the Boltzmann distribution are −55±5 mV and −18 mV, respectively.

#### Activation kinetics

The apparent activation time constants (τ_act_) of HERG channels were determined by the single exponential fit to the step I_HERG_ during depolarizing pulse to 0 mV. The values ranged from 330 ms to 50 ms between voltage steps of −20 mV and +20 mV (Fig. 2B).

#### Deactivation kinetics

To evaluate the voltage-dependence of the apparent deactivation time constants (τ_deact_) of HERG channels, the voltage protocol as shown in Fig. 2B was applied. The decaying phase of the tail I_HERG_ elicited at various hyperpolarizing/repolarizing steps (from −120 mV to −50 mV), following a depolarizing prepulse to +40 mV, were fit by the single exponential to obtain τ_deact_. It is important to note that apparent deactivation process was absent at potentials positive to −50 mV, and minor and slow deactivation was seen at potentials from −50 mV to −70 mV with time constants of 3.8 s to 1.4 s.

#### Inactivation kinetics

The inactivation kinetics (τ_inact_) was analyzed with the voltage protocol shown in the inset of Fig. 2C. The decaying tail currents recorded upon depolarization to various potentials ranging from −80 mV to +40 mV to inactivate HERG channels, preceded by a 10-ms hyperpolarizing prepulse to −120 mV to let channels fully recover, were fit to the monoexponential function and the values of τ_inact_ ranged from 8.5 ms to 1.3 ms between −80 mV and +40 mV.

#### Reactivation kinetics

The reactivation time course (τ_react_) was determined with the same voltage protocol as that for steady-state inactivation. The monoexponential fit was applied to the activating phase of the tail I_HERG_ evoked at various hyperpolarizing/repolarizing steps following the inactivation steps at +40 mV. The reactivation time constants (τ_react_) range from 8.2 ms to 1.2 ms for voltages from −120 mV to −40 mV.

These results indicate that (1) with respect to voltage, HERG channel inactivated from more negative potentials than it activates (−55 mV *vs*. −30 mV) and (2) with respect to time, the time courses of inactivation and reactivation from inactivation were comparable, and were 1∼2 orders of magnitude faster than the activation and 3 orders of magnitude faster than the deactivation processes within the physiological voltages spanned by an AP.

### General Characteristics of I_HERG_ Assessed by Action Potential Waveforms from Different Areas of the Heart

**Figure 3:**
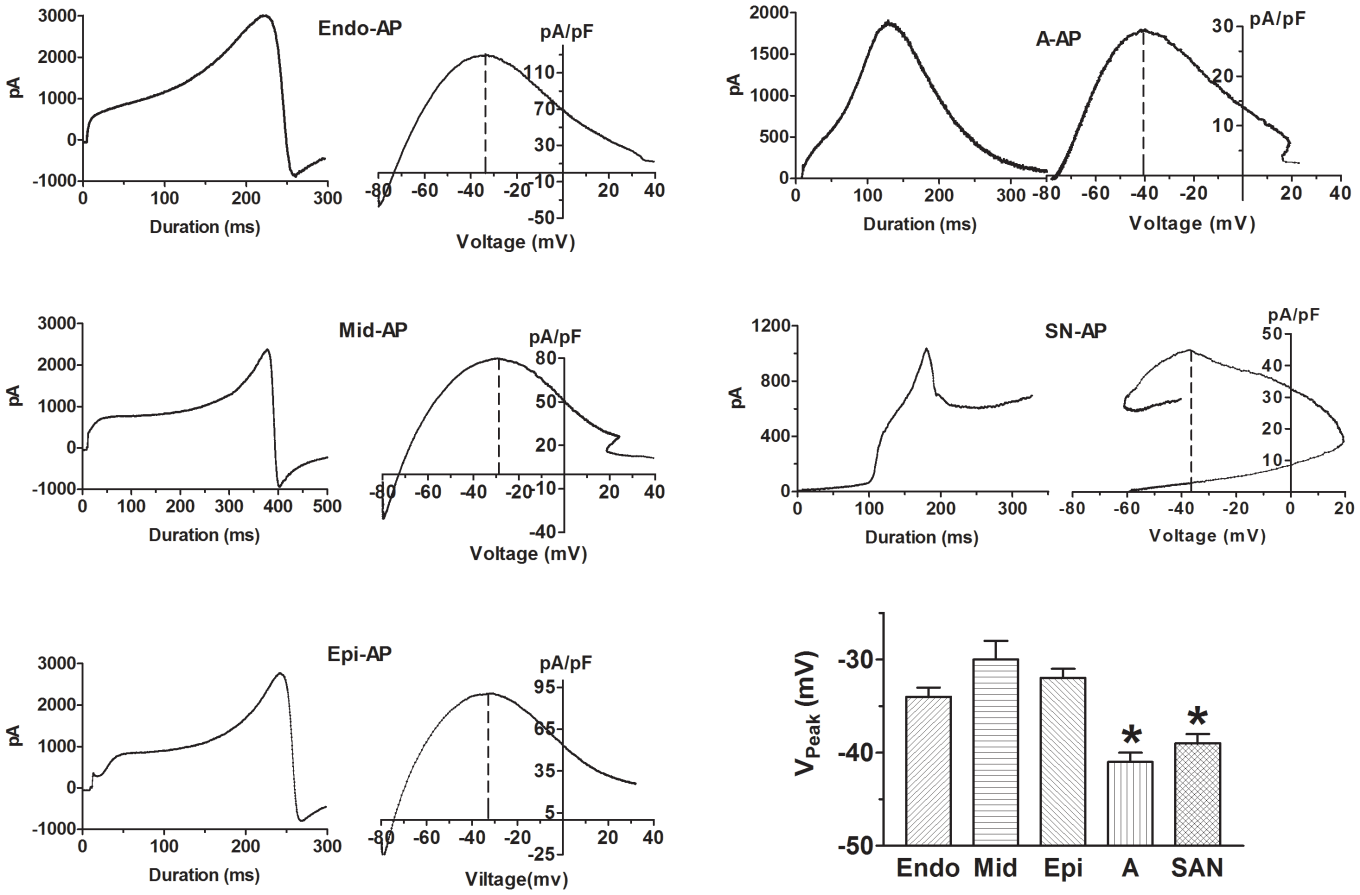
I_HERG_ waveforms and I–V relationships measured by AP-clamp. Left panels: Typical examples of current waveforms recorded by AP-clamp; Right panels: I–V relationships averaged from at least 15 cells. Dash lines indicate the voltages at which I_HERG_ peaks (V_Peak_). The mean V_Peak_ was averaged from 15 cells for each group. *p<0.05 *vs.* Endo.

On the basis of our data with conventional square pulse protocols, we predicted that the activation of HERG is rapid during an AP. However, due to rapid inactivation relatively little current flows until the potential becomes less positive than 0 mV. The removal of inactivation then allows more current to flow, giving rise to the distinct pattern of I_HERG_ changes during the AP. I_HERG_ elicited by AP-clamp could be roughly divided into 2∼3 rising phases and 2 decaying phases. The initial rising phase was rapid and corresponded to the phase 0 upstroke of AP waveform. This was followed by a slowly rising phase corresponding to the phase 2 plateau and a subsequent fast rising phase during phase 3 repolarization, with ventricular AP waveforms (V-APs). With atrial AP (A-AP) and SAN-AP waveforms, the second slowly rising phase was absent presumably because of the short phase 2 plateau duration. After reaching the peak, I_HERG_ rapidly decayed during the final repolarization. Subsequent to the fast decay was a slowly decaying phase occurring after complete repolarization, equivalent to the tail I_HERG_ recorded upon repolarization steps (Fig. 3). Note that there was an inward tail at the end of the trace after complete repolarization of V-APs. This was likely due to the fact that the resting membrane potential of our cells was around −74 mV and repolarizing to −80 mV with V-APs generated inward current, whereas repolarizing to −70 mV with A-AP or to −55 mV with SAN-AP produced outward tail currents.

**Figure 4:**
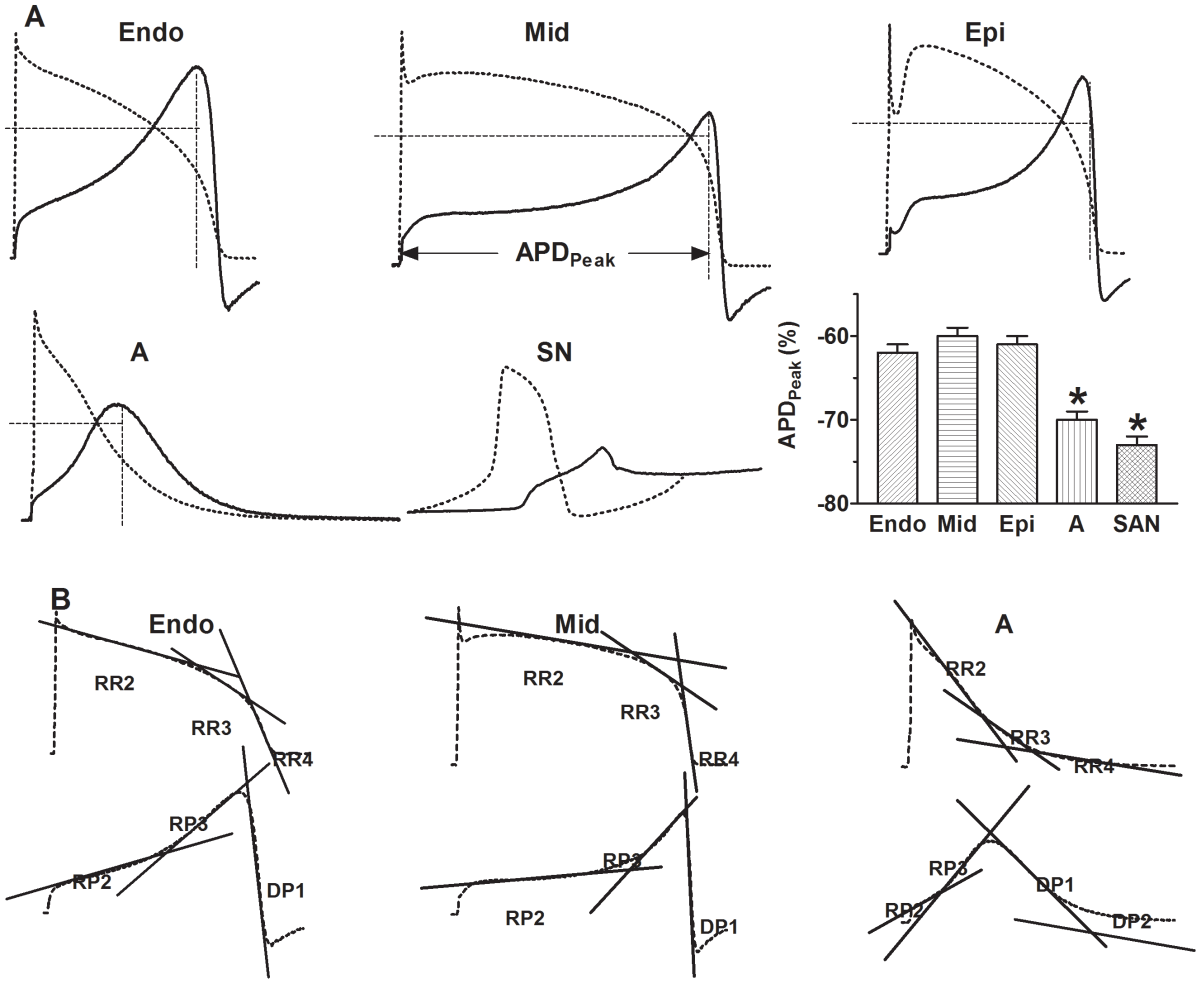
Direct comparison between AP of various regions and the corresponding I_HERG_ waveforms. ***A***, Superimposed AP (dotted line) and I_HERG_ (solid line) traces. Vertical dash lines indicate APD at which I_HERG_ peaks (APD_Peak_). Note that the horizontal dash lines pass through the converging point between AP and I_HERG_ and form the boundary for the mirror-image relationship between AP and I_HERG_. The mean data of APD_Peak_ were averaged from 15 cells for each group. The amplitude and duration scales are the same for all recordings. *p<0.05 *vs.* Endo. ***B***, AP and I_HERG_ traces showing the measurements of slopes of various phases by linear regression indicated by the straight lines, as a measure of rate of repolarization for AP and rising/decaying phase for I_HERG_. RR2, RR3 and RR4, repolarization rate corresponding to the phases of APD_50_, APD_50_-APD_60_ and APD_60_-APD_90_, respectively. RP2 and RP3: rising phase 2 and 3 of I_HERG_ trace, respectively; DP1 and DP2: decaying phase 1 and 2 of I_HERG_ trace, respectively.

Detailed analysis of AP-clamp revealed several novel aspects of I_HERG_ important to our understanding of HERG function and biophysics, which would otherwise be masked by the conventional step protocols. First, although the I–V relationship of AP-clamp I_HERG_ was bell-shaped, similar to the step I_HERG_, the potentials at which I_HERG_ peaked were around 20 mV more negative with AP-clamp than with conventional step clamp. Specifically, I_HERG_ peaked at −10 mV with pulse protocols, but at −30 to −40 mV with AP waveforms (Fig. 3 and Table 2). With respect to APD, I_HERG_ peaked at the time when membrane potential reached 60–70% full repolarization, that is, APD_60_ and APD_70_ (Fig. 3 and Table 2). For more straightforward view of these relationships, direct comparison between AP configuration and I_HERG_ waveform is depicted in Fig. 4A. The vertical dash lines indicate the position where I_HERG_ peaks in relation to APD. Note that the amplitude and duration scales are the same for all recordings in Fig. 4A and the I_HERG_ of Endo-AP peaked to the highest level, while the I_HERG_ of SAN-AP peaked to the lowest level, relative to the respective AP amplitudes (horizontal dash lines).

**Table 2:** Characteristics of I_HERG_ Reported by AP-Clamp.

	Endo-AP	Mid-AP	Epi-AP	A-AP	SAN-AP
**V_Peak_ (mV)**	−34±1	−30±2	−32±1	−41±1	−39±0
**APD_Peak_ (% Repolarization)**	62±1 (APD_62_)	60±2 (APD_60_)	61±1 (APD_61_)	70±1 (APD_70_)	73±1 (APD_73_)
***A*** (pA/pF*s×1000)	−17±1	−15±1	−11±1	−11±1	−6±1
***V*** **_1/2,IT_/** ***k*** ** (mV)**	−37/19	−50/28	−51/26	−38/14	N/A
**RP2 (pA/ms)**	21±1	8±0	13±1	35±1	22±2
**RP3 (pA/ms)**	45±3	50±3	50±3	55±4	26±2
**DP1 (pA/ms)**	−80±4	−85±5	−32±1	−50±3	−45±5
**DP2 (pA/ms)**	N/A	N/A	N/A	−12±1	N/A

**V_Peak_**: the voltage at which I_HERG_ peaks; **APD_Peak_**: % repolarization at which I_HERG_ peaks; hence, the value 62% in the table can be expressed as APD_62_; ***A***: area under I_HERG_ trace, a measure of the volume of K^+^ passing through HERG channel during an AP; ***V*** **_1/2,IT_**: voltage for half maximum inactivation calculated from the Boltzmann fit to the instantaneous inactivation curve; ***k***: sloe factor from the Boltzmann fit for instantaneous conductance curve; **RP2** and **RP3**: rising phase 2 and 3 of I_HERG_ trace, respectively; **DP1** and **DP2**: decaying phase 1 and 2 of I_HERG_ trace, respectively. Positive sign indicate ascending and negative sign descending directions.

Second, to quantitatively describe the time-dependent changes of I_HERG_ during an AP waveform, we calculated the slope (or the rates) of various rising and decaying phases by linear regression to the data points spanning the steepest portion of the given phases (Fig. 4B). The corresponding phases of APD were also analyzed for the rates of repolarization by the same methods (Fig. 4B). The resulting slopes were used to measure the rates of ascending and descending phases of I_HERG_. Comparison between the rates of I_HERG_ changes and the rates of various phases of AP waveforms revealed that the rates of I_HERG_ rising phases and decaying phase depended upon the rates of corresponding phases of membrane repolarization. That is, the greater the rate of repolarization (the steeper of the phase) of an AP was, the faster the rising (or decaying) phase of I_HERG_ was. This point was further reinforced by the fact that when the AP and the corresponding I_HERG_ trace were superimposed, the repolarizing phases of the AP and the rising phases of the I_HERG_ had a nearly perfect mirror-image relationship (Fig. 4A).

**Figure 5:**
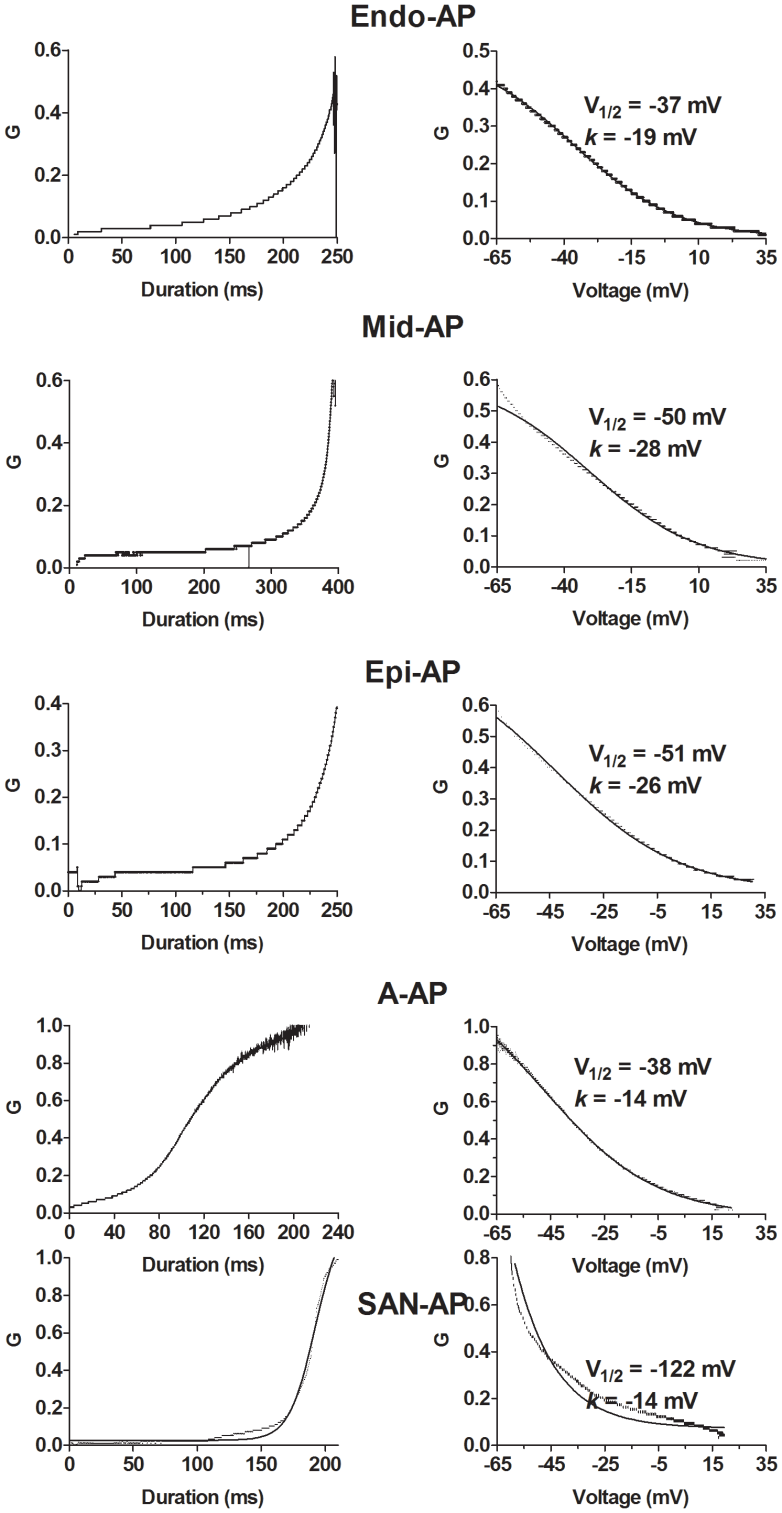
Time- and voltage-dependence of the instantaneous conductance of HERG K^+^ channels reported by AP-clamp. The instantaneous conductance (G) was calculated by dividing the current amplitude by the membrane potential of an AP. Left panels: G as a function of time (APD); Right panel: G as a function of membrane potential. The latter is considered as instantaneous inactivation. The lines represent the Boltzmann fit to the experimental data points.

Third, both the voltage-dependence and time-dependence of changes of the instantaneous conductance of HERG channels (*G*) were analyzed according to the equation *G* = I/V (where I represents a given I_HERG_ amplitude at the corresponding voltage (*V*) during AP-clamp). As shown in Fig. 5, the *G*-*V* relationship with V-APs and A-AP was sigmoidal and can be fit by the Boltzmann function, and the *G*-*V* relationship with SAN-AP waveform was exponential. The *G*-*V* relationship could be viewed as an instantaneous inactivation curve with conductance or channel availability decreasing as membrane depolarizes or as an instantaneous reactivation curve with conductance or channel availability increasing as membrane repolarizes; with respect to AP waveform, the latter might be more informative. The resulting instantaneous *V*_1/2_ values for Epi-AP (−51 mV) and Mid-AP (−51 mV) were similar to that determined by pulse protocols and for Endo-AP (−37 mV) and A-AP (−38 mV) the values corresponded to more positive potentials. By comparison, the *k* values for Endo-AP (−18 mV) and A-AP (−14 mV) were close to that with pulse protocols and those for Epi-AP (−28 mV) and Mid-AP (−26 mV) were much shallower. When G_in_ was expressed as a function of time (*T*) or duration, we obtained the instantaneous *G*-*T* relationship of HERG channels. The *G*-*T* relationship showed exponential increases with *V*-APs while sigmoidal increases with A-AP and SAN-AP waveforms.

**Figure 6:**
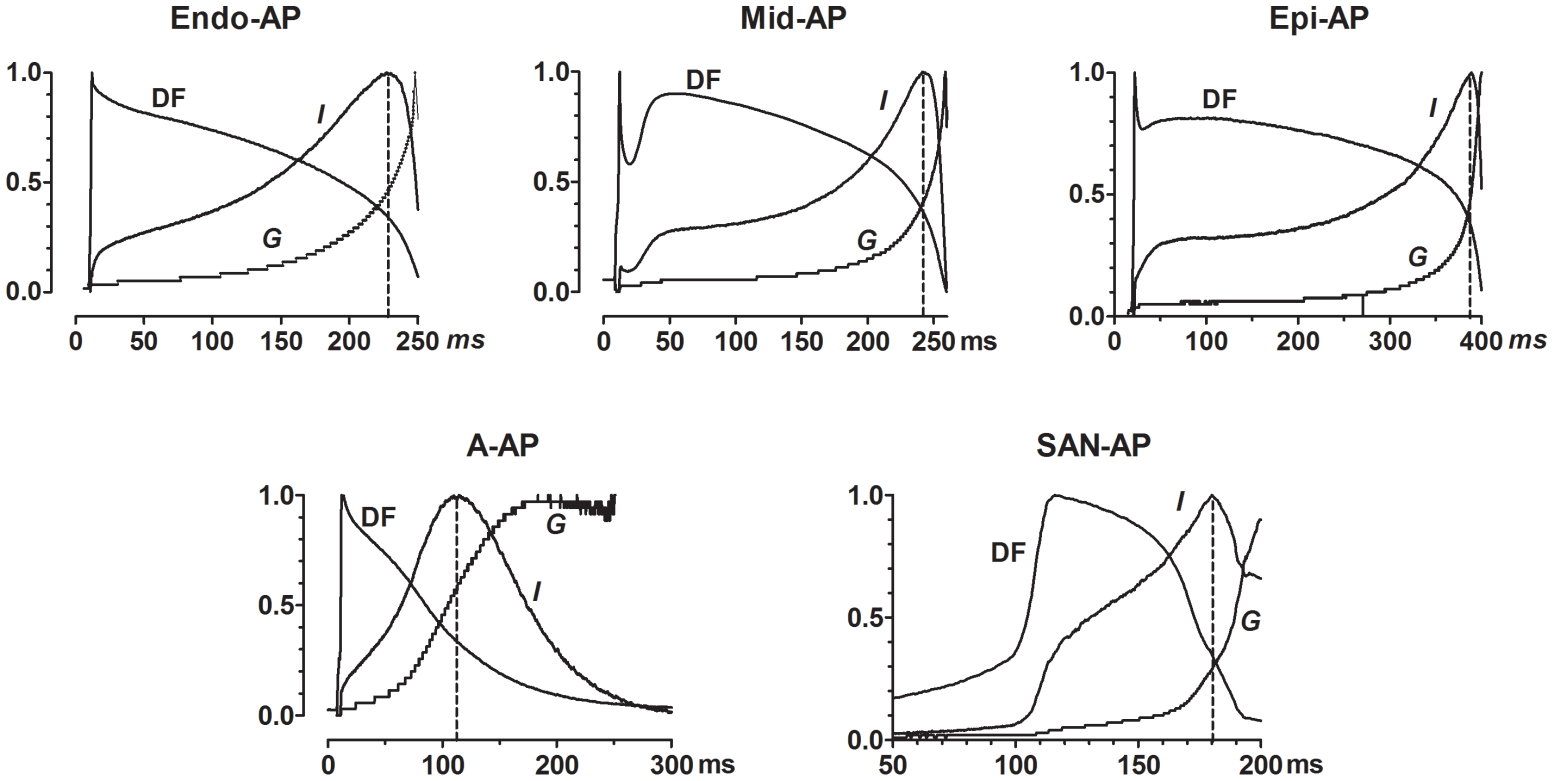
Direct comparisons of normalized current (*I *), conductance (*G*) and driving force (DF). G and DF were calculated using the equations: *G* = *I*/*V* and DF = *I*/*G*, respectively, where *V* represents the dynamic membrane potential during an AP. All data points were normalized by dividing by the maximum values. The vertical dash lines define the time at which I_HERG_ peaks. Note that while *I* begins to decline, *G* continues to rise, after the peak.

Finally, as already described above, I_HERG_ peaked at around APD_60_ and APD_70_ with respect to APD and at between −30 mV and −40 mV with respect to membrane potential. Evidently, the rising phase of I_HERG_ was mainly due to reactivation of HERG channels during repolarization from inactivation rendered upon initial depolarization. However, it was unclear what determines the decaying phases of I_HERG_ during AP-clamp. One possibility is deactivation of HERG channels as commonly believed [21], [23], [27]. Nonetheless, close inspection of deactivation process with step protocol revealed that the deactivation time course was slow and virtually absent at potentials positive to −50 mV (Fig. 2B), implying that at the voltages where I_HERG_ started to decline, no channel deactivation occurred. It is therefore unlikely that the decaying of I_HERG_ during an AP is ascribed to deactivation process. To resolve the issue, we calculated the instantaneous driving forces (DF) during an AP waveform by subtracting the voltages at given time points from the reversal potential of I_HERG_ in a given cell. DF and *G* were then normalized to their own maximum values. The predicted I_HERG_ amplitude was determined by multiplying DF by *G* (*I* = *G*×DF). As shown in Fig. 6, the values were maximal at around −30 mV in voltage and APD_60_ in duration for V-APs and −40 mV and APD_70_ for A-AP and SAN-AP. Noticeably, at durations after APD_60_ and APD_70_, *G* continually increased while DF began to reduce. The data suggest that the rapid decaying phase of I_HERG_ with AP-clamp was a consequence of falling of DF.

### Spatial Heterogeneity of I_HERG_ Revealed by AP-Clamp

Comparison of AP-clamp I_HERG_ revealed some important regional differences of HERG function in terms of its voltage-dependent and time-dependent properties.

First, I_HERG_ with V-APs from three different layers all peaked at approximately −30 mV and with A-AP and SAN-AP at around −40 mV (Fig. 3 and Table 2).

Second, I_HERG_ elicited with V-APs peaked at duration equivalent to APD_60_, despite the transmural differences in the length of APD_60_. By comparison, I_HERG_ recorded with A-AP or SAN-AP peaked at duration to APD_70_ (Fig. 4 and Table 2). These corresponded to 219 ms, 378 ms and 245 ms for APD_60_ of Endo-AP, Mid-AP and Epi-AP, respectively, and 135 and 80 ms for APD_70_ of A-AP and SAN-AP, respectively.

**Figure 7:**
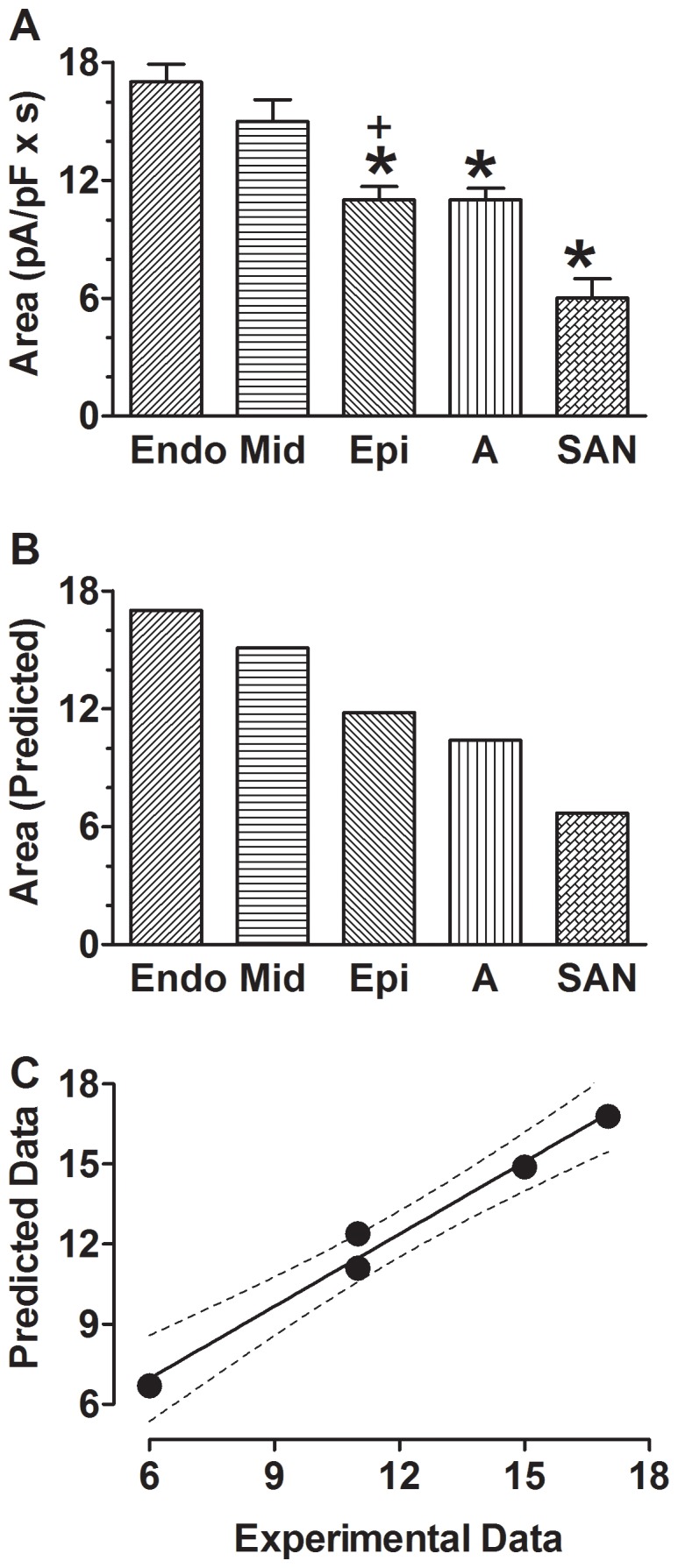
Spatial and transmural heterogeneity of HERG K^+^ channel function. ***A***, Experimental data of the area under I_HERG_ trace, a quantitative index of the size of current flow through HERG channels during action potentials. *p<0.05 *vs.* Endo; +*p*<0.05 F-test (ANOVA) for transmural difference. ***B***, Theoretical (Predicted) data of the area under I_HERG_ trace. ***C***, Correlation between experimental data and predicted data. The solid line represents the linear regression to the data points and the dotted lines indicate 95% confidence.

To better quantify the size of I_HERG_, we calculated the area covered by I_HERG_ trace elicited by an AP using the integration function of Graphpad Prism software and setting the baseline at the zero current level; such analysis gives more accurate measure of K^+^ flux carried by HERG channels during an AP. I_HERG_ determined in this way demonstrated spatial differences and as expected, the area was in general greater in the region with longer APD. For instance, the length of APD was in the order of V-APD>A-APD>SAN-APD, and correspondingly, the size of I_HERG_ area was also in the same order (Fig. 7A).

### Transmural Heterogeneity of I_HERG_ Revealed by AP-Clamp

Unexpectedly, when comparing I_HERG_ elicited with AP waveforms across the left ventricular wall, the area, in the order of Endo-AP>Mid-AP>Epi-AP (Fig. 7A), did not correlate with the length of APD alone. Apparently, other factors in addition to APD also play an important role in determining the size of I_HERG_ area. As already mentioned above, HERG reactivation or reopening depended upon the rate of membrane repolarization and DF determines the size of I_HERG_. This implies that the rate of repolarization (a measure of HERG conductance *G*) and DF during various phases of an AP should be taken into consideration for understanding the spatial heterogeneity of I_HERG_ area. We then formulated the following equations to predict relative size of I_HERG_ area of different regions:
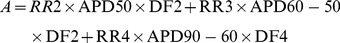
(1)

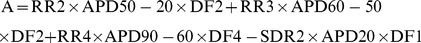
(2)

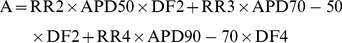
(3)where *A* represents the area of I_HERG_, RR the rate of repolarization of the given phase of an AP as specified by the number, and DF the mean driving force of the given phase of an AP as specified by the number. APD_50_ approximates the length of the plateau phase, ΔAPD_60-50_ (APD_60_-APD_50_) the duration of transition from phase 2 to phase 3 repolarization, and ΔAPD_90-60_ (APD_90_-APD_60_) the duration phase 3 repolarization. SDR2 indicates the rate of secondary depolarization and APD_20_ approximates the duration of the secondary depolarization, which were seen with Epi-AP because of the spike-and-dome morphology. Equation 1 was used for Endo-AP and Mid-AP, equation 2 for Epi-AP, and equation 3 for A-AP and SAN-AP for I_HERG_ peaks at APD_70_, instead of APD_60_ like Endo-AP, Epi-AP and Mid-AP, in these cells. Note that SDR2 was negative for depolarization. Using the values listed in Table 1, we calculated the *A* values for Endo-AP, Mid-AP and Epi-AP (Fig. 7A), respectively, which are in agreement with the order of I_HERG_ area (Fig. 7B). Interestingly, when the equation was applied to A-AP and SAN-AP, the resulting *A* values also well reflect the relative size of I_HERG_ area in these regions. This notion was further reinforced by the close correlation between the predicted and experimental I_HERG_ areas (Fig. 7C).

### Alterations of I_HERG_ in Response to Blockade of I_to_, I_Kur_, or I_Ca,L_

**Figure 8:**
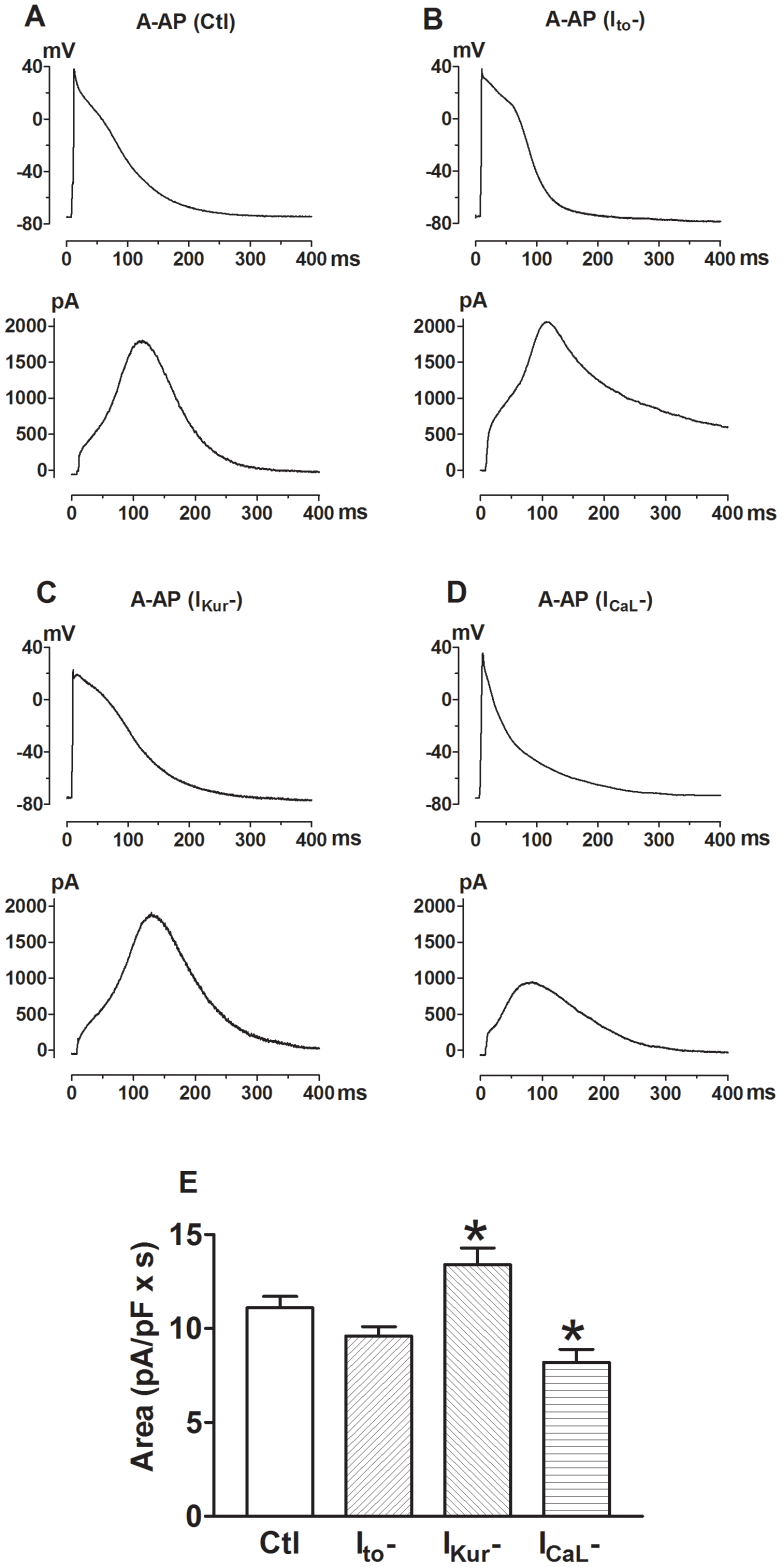
Influence of selective blockade of ion currents activating prior to I_Kr_ on I_HERG_ function reported by AP-clamp with atrial AP waveforms. ***A*–*D***, A-AP configurations in the presence of selective blockers to inhibit I_to_, I_Kur_ or I_CaL_ from canine atrial myocytes, used to record the respective I_HERG_ waveforms. E, Mean data (n = 18 cells for each group) of area under I_HERG_ traces. I_to_–: I_to_ blocked by 500 nM nicotine; I_Kur.d_–: I_Kur.d_ blocked by 100 nM 4-AP; I_CaL_–: I_CaL_ blocked by 10 µM nisodipine. *p<0.05 *vs.* Ctl.

**Figure 9:**
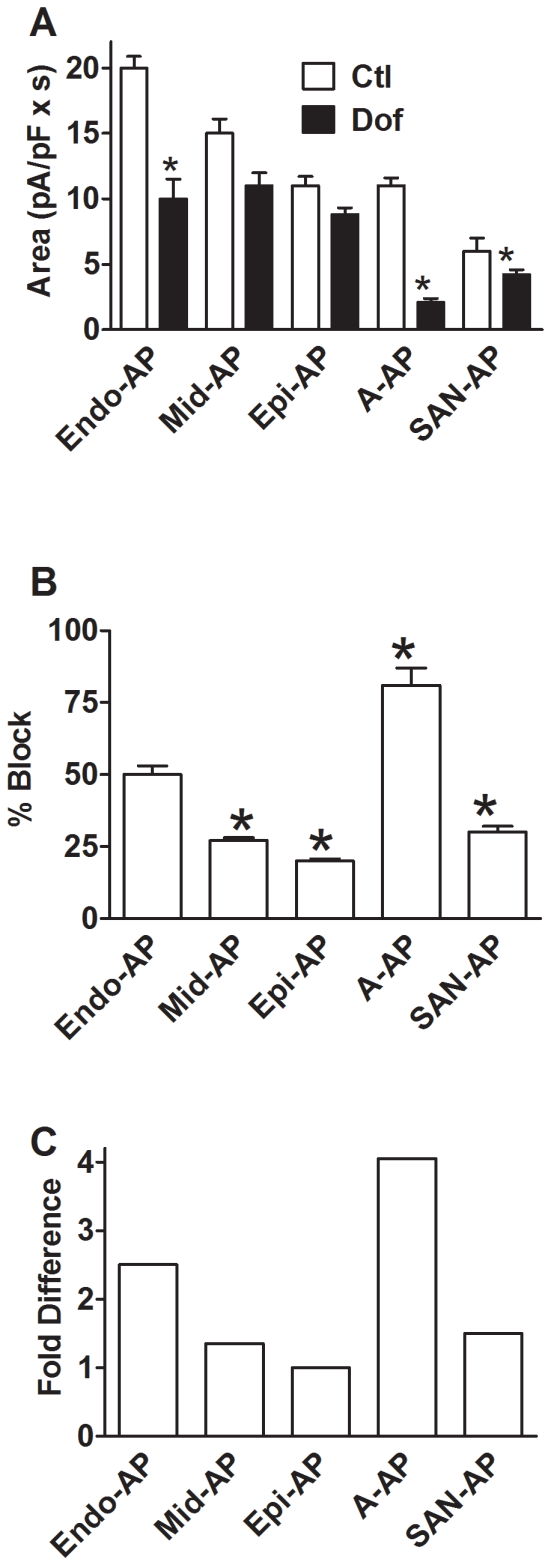
Pharmacological property of I_HERG_ reported by AP-clamp. ***A***, effects of I_Kr_/HERG blocker dofetilide (Dof, 10 nM) on I_HERG_ with AP waveforms of various regions. *p<0.05 *vs.* Ctl (n = 18 cells). ***B***, % block of I_HERG_ over control by dofetilide with AP waveforms of various regions. *p<0.05 *vs.* Endo-AP (n = 18 cells). ***C***, Fold difference of I_HERG_ blockade by dofetilide with AP waveforms of various regions. Data were normalized to the value with Epi-AP.

Ion currents activate in a certain sequence during an action potential. The one that activates in earlier phases may affect activation of the ones that activate in later phases by setting the voltage and time preconditions for the channel state. For example, the transient outward K^+^ current (I_to_) activates in the early phase of an AP and alteration of I_to_ may change the function of the subsequently activated currents such as I_Kr_ by altering AP morphology (voltage and duration). In a previous study, the authors showed that nicotine selectively inhibits I_to_ at low concentrations without affecting other K^+^ currents [28]. To investigate the potential influence of I_to_ alteration on I_Kr_, we recorded APs in the presence of nicotine (500 nM) to inhibit I_to_ in canine atrial myocytes and these APs have elevated plateau level and markedly shortened APD. The ultrarapid delayed rectifier K^+^ current (I_Kur_) is an atrium-specific and activates in the early phase of AP with partial inactivation, contributing to the plateau repolarization of atrial myocytes [25], [29]. It is highly sensitive to 4-aminopyridine (4-AP) block, with micromolar concentrations of 4-AP nearly abolishing I_Kur_ without significantly affecting other ion currents and blockade of I_Kur_ produces some 50–60% APD lengthening. Here the APs recorded from canine right atrial cells in the presence of 100 µM 4-AP to block I_Kur.d_ have elevated plateau level and prolonged APD. The characteristics of L-type Ca^2+^ current (I_CaL_) blockade by 10 µM nisodipine is the loss of plateau phase and shortening of APD, particularly the early phase repolarization (Fig. 8). The I_HERG_ elicited by the AP waveforms recorded under these different conditions demonstrated similar but different characteristics, as shown in Fig. 9. I_HERG_ peaked at 110 ms under control conditions and when I_to_ was inhibited (labeled I_to_–). When I_Kur_ was blocked (labeled I_Kur_–), the peak of I_HERG_ shifted to 140 ms, whereas when I_CaL_ was blocked (labeled I_CaL_–) I_HERG_ peaked sooner at 75 ms. In respect with voltage, the maximum I_HERG_ occurred at around −40 to −43 mV under control conditions or with I_Kur_– or I_CaL_–, and it was some 10 mV more negative shift when I_to_ was inhibited. The most notable difference was the size of I_HERG_ area. The characteristics of the atrial APs used for AP-clamp and the corresponding I_HERG_ in the presence of varying K^+^ channel blockers to inhibit I_to_, I_Kur_ or I_CaL_, separately, are summarized in Table 3.

**Table 3:** Characteristics of the Atrial APs Used for AP-Clamp and the Corresponding I_HERG_ in the Presence of Varying K^+^ Channel Blockers to Inhibit I_to_, I_Kur_ or I_CaL_.

	Ctl	I_to_–	I_Kur.d_–	I_CaL_–
**APD_10_ (mV—ms)**	27—17	13—34	26—25	25—15
**APD_20_ (mV—ms)**	16—30	3—60	15—51	14—21
**APD_50_ (mV—ms)**	−19—82	−28—107	−20—86	−20—46
**APD_60_ (mV—ms)**	−30—96	−38—122	−32—93	−31—56
**APD_90_ (mV—ms)**	−64—179	−68—225	−68—142	−64—192
**V_Peak_ (mV)**	−41±1	−43±1	−49±1	−40±1
**APD_Peak_ (% Repolarization)**	−70±1	−65±1	−74±1	−69±1
***A*** (pA/pF*s×1000)	11.1±0.6	9.6±0.5	13.4±0.9	8.2±0.7
***V*** **_1/2,IT_ (** ***k*** **) (mV)**	−38 (19)	−47 (28)	−43 (26)	−38 (14)

I_to_–: I_to_ blocked by 500 nM nicotine; I_Kur.d_–: I_Kur.d_ blocked by 100 nM 4-AP; I_CaL_–: I_CaL_ blocked by 10 µM nisodipine; **V_Peak_**: the voltage at which I_HERG_ peaks; **APD_Peak_**: % repolarization at which I_HERG_ peaks; hence, the value 62% in the table can be expressed as APD_62_; ***A***: area under I_HERG_ trace, a measure of the volume of K^+^ passing through HERG channel during an AP; ***V*** **_1/2,IT_**: voltage for half maximum inactivation calculated from the Boltzmann fit to the instantaneous inactivation curve; ***k***: sloe factor from the Boltzmann fit for instantaneous conductance curve.

### Regional Differences of I_HERG_ in Response to Dofetilide Blockade Revealed by AP-Clamp

Since the effect of I_Kr_/I_HERG_ blockers is state-dependent with most of them generally acting on the open channels and since there is regional difference of APs in terms of the repolarization rate thereby the rate of HERG reopening, we reasoned that the effects of I_Kr_/I_HERG_ blockers are different in cells from different regions of the heart. To test this notion, we went on to study the effects of dofetilide on I_HERG_ using AP-clamp with AP waveforms collected from various regions. As illustrated in Fig. 9, 10 nM dofetilide which produced approximately 50±3% inhibition of I_HERG_ area with Endo-AP reduced the current by 27±1%, 22±1%, 81±6%, and 30±2% with Mid-AP, Epi-AP, A-AP and SAN-AP, respectively (Fig. 9A and 9B). When the data were normalized to Epi-AP, it was clear that the degree of blockade of I_HERG_ with A-AP was around 4-times greater than with Epi-AP and about 2∼3-times greater than in APs of other regions (Fig. 9C). Also important is that there were transmural differences of I_HERG_ in response to dofetilide block. For example, the blockade of I_HERG_ with Endo-AP was 2.5-fold greater than with Epi-AP and 2-fold greater than with Mid-AP (Fig. 9C). Noticeably, these differential effects of dofetilide caused a remarkable change in the pattern of transmural difference of I_HERG_; the normal I_HERG_ gradient was changed to Mid-AP>Epi-AP≥Endo-AP by dofetilide.

## Discussion

### Novel Properties of HERG Channel Reported by AP-Clamp

The present study with AP-clamp revealed several important features of HERG K^+^ channel function, which are otherwise masked with the conventional voltage pulse protocols. Some of these features have been described by previous work from other laboratories using AP-clamp techniques and therefore they will not be repeated in Discussion. Below are the selected aspects which we believe to represent the novel findings on HERG function revealed by the present study.

1. With AP-clamp, I_HERG_ in ventricular cells peaks at around −30 mV (V_Peak_) and in atrial and SAN cells at −40 mV, with respect to voltage, as revealed by the instantaneous I–V relationships (Fig. 3). By comparison, I_HERG_ peaked at −10 mV with conventional pulse protocol. This implies that pulse protocols introduced ∼20–30 mV voltage error to measurement of the voltage-dependence of HERG channel. The fully activated I–V relationship, obtained by various reactivating (repolarizing) steps following an inactivating (depolarizing) pulse with conventional pulse protocols or by ramp protocols, demonstrated the maximum I_HERG_ at −30 mV [21], [23], approximating the AP-clamp value. Unfortunately, the protocol could not distinguish the regional difference of V_Peak_ or the voltage-dependence of HERG channel, especially the difference between V-APs and A-AP.
2. AP-clamp revealed that with respect to APD, I_HERG_ in ventricular cells peaked near APD_60_ and in atrial and SAN cells it peaked near APD_70_ (APD_Peak_). The data imply that APD_Peak_ is predicable regardless of the length of APD. For example, the length of Mid-AP (APD_90_) was around 400 ms and that of Endo-AP and Epi-AP was approximately 250 ms, but I_HERG_ in these regions all peaks at APD_60_ that corresponded to the length of phase 2 plateau. This was consistent with the view that I_Kr_ serves to terminate the phase 2 plateau of APs. Conventional pulse protocols do not provide information concerning APD_Peak_.
3. The initial rising phase (phase 1) of I_HERG_ was elicited by the depolarization phase of an AP and the major rising phases of I_HERG_ (phase 2 and phase 3) corresponded to the repolarization phases of an AP. The rate of rising phase was determined by the rate of membrane repolarization. No such conclusion can be drawn with conventional pulse protocols.
4. There was transmural heterogeneity of I_HERG_ function as indicated by the differences in I_HERG_ area, a measure of total K^+^ passing through HERG channels during an AP. The size of I_HERG_ area was found to be in the order of Endo-AP>Mid-AP>Epi-AP. Conventional pulse protocols would mask this important property of HERG channel.
5. There was regional difference of I_HERG_ in response to dofetilide blockade. Our results demonstrated that the effects of dofetilide on I_HERG_ elicited with APs from different regions of the heart were quantitatively different. The % blockade of I_HERG_ with A-AP was some 4-fold greater than Epi-AP and 2–3 fold greater than APs of other regions. The differences also existed among different layers of left ventricular wall with significantly greater inhibition seen with Endo-AP than with Mid-AP and Epi-AP. Importantly, this transmural difference of I_HERG_ blockade by dofetilide broke the normal pattern of transmural gradient of I_HERG_ from Endo-AP>Mid-AP>Epi-AP under control conditions to Mid-AP>Epi-AP≥Endo-AP in the presence of dofetilide. And the maximum transmural difference was also diminished from 6 (Endo-AP minus Epi-AP) to 2.5 (Mid-AP minus Endo-AP). Obviously, these differences cannot be revealed by using conventional pulse protocols.
6. During an AP, the complex nature of the interactions between different currents means that small changes in one current could alter the behavior of others. Indeed, our data show that I_HERG_ function was influenced by alterations of preceding currents. Our data show that I_HERG_ function varied in response to selective inhibition of any given current activated prior to I_Kr_. For instance, blockade of I_Kur_ markedly enhanced I_HERG_ and in contrast, blockade of I_CaL_ caused pronounced reduction of I_HERG_ (Fig. 8).

### Biophysical Mechanisms for HERG Channel Reported by AP-Clamp

Evidently, HERG function is determined by HERG properties and AP characteristics; the latter indicates the differences in the rate of repolarization that governs the gating of HERG channels.

1. The dynamic changes of I_HERG_ (or the rising phase) during an AP were determined by rapid reactivation or removal of inactivation of HERG channels, a process depending upon the dynamic changes in rate of repolarization.
2. Voltage-dependent rapid reactivation of I_HERG_ determined the regenerative (all-or-none) repolarization. Under normal conditions, the cardiac repolarization process, once initiated, will carry on itself until it reaches full repolarization. The present study provides an explanation for the regenerative repolarization process: Membrane repolarization initiated by I_to_ activation and I_Ca,L_ inactivation tends to reactivates I_HERG_, and I_HERG_ reactivation in turn further repolarizes membrane which results in more I_HERG_ reactivation and further more repolarization.
3. The decaying phase of I_HERG_ with AP-clamp has been thought to be the balance between reactivation and deactivation of HERG channels [23], [30]. Our data, however, provide an alternative mechanism for that. According to our data in Figure 2 as well as previous studies, no apparent deactivation process was seen at potentials positive to −50 mV and slow deactivation was noted at −60 mV to −80 mV with time constants of over 1 s. Considering that the decaying phase of I_HERG_ during a ventricular AP started from −40 mV and spanned <50 ms in duration, it is unlikely that deactivation process was involved. Similarly, changes in HERG conductance did not account for the decline either because during the whole decaying phase of I_HERG_, HERG conductance oppositely kept increasing (Fig. 6). In sharp contrast, the fall of driving force paralleled the declining of I_HERG_. Thus, driving force or the electrochemical gradient may be the major factor determining I_HERG_ decay after the peak. This notion let us speculate that the rapid fall in driving force terminated repolarization and prevented excessive cardiac hyperpolarization that could otherwise happen with regenerative HERG reactivation during repolarization.
4. We used the area covered by I_HERG_ trace to quantify the size of I_HERG_ because it is a more accurate measure of K^+^ efflux through HERG channels during the whole period of an AP. By comparison, I_HERG_ amplitude determined by the peak value only represents the size of I_HERG_ at that particular time point. Due to differences of AP morphology or of the rate of membrane repolarization, I_HERG_ amplitude at any time point was different; changes in membrane potential during an AP were dynamic and so was the I_HERG_ amplitude. To integrate the dynamics, measuring the area is a better method for defining HERG function. Indeed, with the use of area, we were able to identify the regional and transmural heterogeneity of HERG function and variation of drug blockade of I_HERG_. We found that the volume of K^+^ carried by HERG channels during an AP, that is the area under I_HERG_ trace, was determined by at least three factors: rate of repolarization, duration of repolarization and driving force. Using these three parameters we were able to predict the relative size of areas of APs from various regions with the pattern consistent with the experimental data: Endo-AP>Mid-AP>Epi-AP≥A-AP>SAN-AP. With I_HERG_ amplitude as a parameter, such regional differences cannot be revealed.

### Potential Implications of the Results

One of the important implications of our findings is that the repolarization-dependent reactivation of HERG channels contributing to the regenerative repolarization process of cardiac cells may help to suppress arrhythmias initiated by early afterdepolarizations and premature beats [11], [23]. I_HERG_ reactivates to the maximum extent at around APD_60_-APD_70_ with respect to APD (APD_Peak_) and at −30 mV to −40 mV with respect to transmembrane potential (V_Peak_), which on one hand can avoid interfering with normal Ca^2+^ entry into the cells to initiate excitation-contraction coupling because I_CaL_ normally peaks at around −10 mV, and on the other hand can prevent excessive or secondary I_CaL_ activation by generating large volume of outward K^+^ flow. This mechanism can serve to counteract any secondary depolarizations falling during this phase, particularly those generated by I_CaL_.

Another important implication of our findings is that there exist regional heterogeneity and transmural gradient of I_HERG_, when assessed with I_HERG_ area. Given that myocytes from different layers of ventricle or from different regions of the heart bear the same I_Kr_ current densities [26], the differences of I_HERG_ area may reflect the true regional heterogeneity and transmural gradient. These regional differences of I_HERG_ may partially account for the regional variations of cardiac repolarization and may have some important pathophysiological implications: being antiarrhythmic under physiological conditions but also being proarrhythmic or arrhythmogenic when in presence of HERG blockers which create transmural dispersion of repolarization. One example of the latter has been documented by Di Diego *et al* [31] who showed that cisapride (a potent blocker of HERG channel) induced transmural dispersion of repolarization and *torsade de pointes* in the canine left ventricular wedge preparation during epicardial stimulation.

Also significant is that the extents of drug blockade of I_HERG_, specifically, the effects of dofetilide on I_HERG_ are different with APs from different regions. Dofetilide at a concentration of 10 nM demonstrates the greatest inhibition of I_HERG_ (as assessed by area) with A-AP, relative to APs from other regions. Intriguingly, dofetilide is currently in clinical use for suppressing atrial arrhythmias. Moreover, in the heart, the contribution of HERG to cardiac repolarization in any given cell type will also depend on the level of channel expression in that cell. A study reported by Gintant [32] suggests that in canine heart I_Kr_ density in atrium is approximately 50% higher than in ventricle. In terms of the transmural differences of dofetilide blockade of I_HERG_, our results show that dofetilide diminishes the maximum transmural differences of I_HERG_ area to 2.5 (Mid-AP minus Endo-AP) from 6 (Endo-AP minus Epi-AP) for control. Unfortunately, we are unable to draw any conclusions from the present study as to whether the reduction of the transmural difference of I_HERG_ will increase or decrease transmural dispersion of repolarization.

Finally, our data revealed for the first time the interactions between I_Kr_/HERG and I_to_, I_Kur_ or I_CaL_ that activates before I_Kr_/HERG does. The important message from these results can be understood as a gate-safeguard mechanism of HERG function for cardiac repolarization. It is known that I_Kur_ activates rapidly upon membrane depolarization with a linear or slightly outward rectifying I–V relationship and stays activated with only partial inactivation during the whole course of an AP [25], [29], indicating that it contributes to various phases of repolarization, particularly the phase 2 plateau. The present study demonstrates that when I_Kur_ is suppressed, I_Kr_/HERG tends to increase. This can serve as a compensatory mechanism or a “repolarization reserve” to minimize the excessive slowing of repolarization due to I_Kur_ blockade. In sharp contrast, when I_CaL_ is depressed, I_Kr_/HERG tends to decrease. This on one hand can allow a longer time for more Ca^2+^ entry for contractile function and on the other hand can limit the excessive acceleration of repolarization consequent to I_CaL_ reduction. Interestingly, I_to_, which contributes mainly to the initial repolarization, when blocked does not significantly alter I_Kr_/HERG that has relatively small contribution to phase 1 repolarization. It is tempting to speculate that in atrial fibrillation when Ca^2+^ channel function is depressed [33], I_Kr_ will decrease accordingly so as to promote Ca^2+^ entry and minimize APD shortening.

### Limitations of the Study

The assessment of I_HERG_ in the present study was based upon a few selected AP waveforms, which are representative of APs in different regions of the heart. However, it should be noted that the results may not be extrapolated to cells from other regions or sub-regions as AP waveforms are different in every different cells. Moreover, in native cells with co-existing multiple ion currents that can influence the function of HERG channels, the properties of I_Kr_ may not be the same as I_HERG_ recorded in a HERG-overexpressing cell line.

## References

[1] Sanguinetti MC, Jurkiewicz NK (1990) Two components of cardiac delayed rectifier K^+^ current. Differential sensitivity to block by class III antiarrhythmic agents. J Gen Physiol 96: 195–215. 2170562 10.1085/jgp.96.1.195 PMC2228985

[2] Wang Z, Fermini B, Nattel S (1993) Sustained depolarization-induced outward current in human atrial myocytes: Evidence for a novel delayed rectifier potassium current similar to Kv1.5 cloned channel currents. Circ Res 73: 1061–76. 8222078 10.1161/01.res.73.6.1061

[3] Wang Z, Fermini B, Nattel S (1993) The delayed outward potassium current (I_K_) in human atrial myocytes. Circ Res 73: 276–85. 8330373 10.1161/01.res.73.2.276

[4] Sanguinetti MC, Jiang C, Curran ME, Keating MT (1995) A mechanistic link between an inherited and an acquired cardiac arrhythmia: HERG encodes the I_Kr_ potassium channel. Cell 81: 299–307. 7736582 10.1016/0092-8674(95)90340-2

[5] Taglialatela M, Castaldo P, Pannaccione A (1998) Human ether-a-go-go related gene (HERG) K^+^ channels as pharmacological targets: present and future implications. Biochem Pharmacol 55: 1741–6. 9714291 10.1016/s0006-2952(98)00002-1

[6] Marban E (2002) Cardiac channelopathies. Nature 415: 213–8. 11805845 10.1038/415213a

[7] Lodge NJ, Normand DE (1997) Alterations in I_to1_, I_Kr_ and I_K1_ density in the BIO TO-2 strain of syrian myopathic hamsters. J Mol Cell Cardiol 29: 3211–21. 9441828 10.1006/jmcc.1997.0548

[8] Tsuji Y, Opthof T, Kamiya K, Yasui K, Liu W, et al (2000) Pacing-induced heart failure causes a reduction of delayed rectifier potassium currents along with decreases in calcium and transient outward currents in rabbit ventricle. Cardiovasc Res 48: 300–9. 11054476 10.1016/s0008-6363(00)00180-2

[9] Priebe L, Beuckelmann DJ (1998) Simulation study of cellular electric properties in heart failure. Circ Res 82: 1206–23. 9633920 10.1161/01.res.82.11.1206

[10] Wang J, Wang H, Han H, Zhang Y, Yang B, et al (2001) The phospholipid metabolite 1-palmitoyl-lysophosphatidylcholine enhances human ether-a-go-go related gene (HERG) K^+^ channel function. Circulation 104: 2645–8. 11723012 10.1161/hc4701.100513

[11] Hua F, Johns DC, Gilmour RF Jr (2004) Suppression of electrical alternants by overexpression of HERG in canine ventricular myocytes. Am J Physiol 286: H2342–51. 10.1152/ajpheart.00793.2003 14962839

[12] Hua F, Robert F (2004) Gilmour RF Jr (2004) Contribution of I_Kr_ to rate-dependent action potential dynamics in canine endocardium. Circ Res 94: 810–9. 14963001 10.1161/01.RES.0000121102.24277.89

[13] Boyden PA, Jeck CD (1995) Ion channel function in disease. Cardiovasc Res 29: 312–8. 7540109

[14] Carmeliet E (1999) Cardiac ionic currents and acute ischemia: from channels to arrhythmias. Physiol Rev 79: 917–1017. 10390520 10.1152/physrev.1999.79.3.917

[15] Wang J, Wang H, Zhang Y, Nattel S, Wang Z (2004) Impairment of HERG K^+^ channel function by tumor necrosis factor-α: role of reactive oxygen species as a mediator. J Biol Chem 279: 13289–92. 14973143 10.1074/jbc.C400025200

[16] Corr PB, Yamada KA, Creer MH, Wu J, Mchowat J, et al.. (1995) Amphipathic lipid metabolites and arrhythmias during ischemia. In Cardiac Electrophysiology: From Cell to Bedside, ed. Zipes DP & Jalife J, pp. 182–203. Philadelphia, WB Saunders.

[17] Goldhaber JI, Deutsch N, Alexander LD, Weiss JN (1998) Lysophosphatidylcholine and cellular potassium loss in isolated rabbit ventricle. J Cardiovasc Pharmacol Ther 3: 37–42. 10684479 10.1177/107424849800300105

[18] Litovsky SH, Antzelevitch C (1989) Rate dependence of action potential duration and refractoriness in canine ventricular endocardium differs from that of epicardium: role of the transient outward current. J Am Coll Cardiol 14: 1053–66. 2551947 10.1016/0735-1097(89)90490-7

[19] Antzelevitch C, Fish J (2001) Electrical heterogeneity within the ventricular wall. Basic Res Cardiol 96: 517–27. 11770069 10.1007/s003950170002

[20] Sicouri S, Antzelevitch C (1991) A subpopulation of cells with unique electrophysiological properties in the deep subepicardium of the canine ventricle. The M cell. Circ Res 68: 1729–41. 2036721 10.1161/01.res.68.6.1729

[21] Anson BD, Ackerman MJ, Tester DJ, Will ML (2004) Molecular and functional characterization of common polymorphisms in HERG (KCNH2) potassium channels. Am J Physiol 286: H2434–41. 10.1152/ajpheart.00891.2003 14975928

[22] Berecki G, Zegers JG, Verkerk AO, Bhuiyan ZA, de Jonge B, et al (2005) HERG channel (dys)function revealed by dynamic action potential clamp technique. Biophys J 88: 566–78. 15475579 10.1529/biophysj.104.047290 PMC1305034

[23] Hancox JC, Levi AJ, Witchel HJ (1998) Time course and voltage dependence of expressed HERG current compared with native “rapid” delayed rectifier K current during the cardiac ventricular action potential. Pflügers Arch 436: 843–53. 9799397 10.1007/s004240050713

[24] Zhang Y, Han H, Wang J, Wang H, Yang B, et al (2003) Impairment of HERG (Human ether-a-go-go Related Gene) K^+^ channel function by hypoglycemia and hyperglycemia: similar phenotypes but different mechanisms. J Biol Chem 278: 10417–26. 12531891 10.1074/jbc.M211044200

[25] Yue L, Melnyk P, Gaspo R, Wang Z, Nattel S (1999) The molecular mechanism of ionic remodelling of repolarization in a dog model of atrial fibrillation. Circ Res 84: 776–84. 10205145 10.1161/01.res.84.7.776

[26] Liu DW, Antzelevitch C (1995) Characteristics of the delayed rectifier current (I_Kr_ and I_Ks_) in canine ventricular epicardial, midmyocardial, and endocardial myocytes. A weaker I_Ks_ contributes to the longer action potential of the M cell. Circ Res 76: 351–65. 7859382 10.1161/01.res.76.3.351

[27] Lu Y, Mahaut-Smith MP, Huang CL, Vandenberg JI (2003) Mutant MiRP1 subunits modulate HERG K^+^ channel gating: a mechanism for pro-arrhythmia in long QT syndrome type 6. J Physiol (Lond) 551: 253–62. 12923204 10.1113/jphysiol.2003.046045 PMC2343156

[28] Wang H, Shi H, Zhang L, Pourrier M, Yang B, et al (2000) Nicotine is a potent blocker of the cardiac A-type K^+^ channels. Effects on cloned Kv4.3 channels and native transient outward current. Circulation 102: 1165–71. 10973847 10.1161/01.cir.102.10.1165

[29] Wang Z, Fermini B, Nattel S (1993) Sustained depolarization-induced outward current in human atrial myocytes: Evidence for a novel delayed rectifier potassium current similar to Kv1.5 cloned channel currents. Circ Res 73: 1061–76. 8222078 10.1161/01.res.73.6.1061

[30] Zhou Z, Gong Q, Ye B, Fan Z, Makielski J, et al (1998) Properties of HERG channels stably expressed in HEK 293 cells studied at physiological temperature. Biophys J 74: 230–41. 9449325 10.1016/S0006-3495(98)77782-3 PMC1299377

[31] Di Diego JM, Belardinelli L, Antzelevitch C (2003) Cisapride-induced transmural dispersion of repolarization and torsade de pointes in the canine left ventricular wedge preparation during epicardial stimulation. Circulation 108: 1027–33. 12912819 10.1161/01.CIR.0000085066.05180.40

[32] Gintant GA (2000) Characterization and functional consequences of delayed rectifier current transient in ventricular repolarization. Am J Physiol 278: H806–17. 10.1152/ajpheart.2000.278.3.H806 10710349

[33] Yue L, Feng J, Wang Z, Nattel S (2000) Effects of the antiarrhythmic agents ambasilide, quinidine, flecainide and verapamil on ultra-rapid delayed rectifier potassium currents in canine atrial myocytes. Cardiovasc Res 46: 151–61. 10727663 10.1016/s0008-6363(99)00430-7

